# Association of Regular Thrombus Surface Phenotype With Complete Recanalization in First-Line Contact Aspiration Thrombectomy for Basilar Artery Occlusion

**DOI:** 10.3389/fneur.2021.666933

**Published:** 2021-09-08

**Authors:** Daniel Kaiser, Pawel Krukowski, Kevin Hädrich, Robert Winzer, Lars-Peder Pallesen, Matthias Gawlitza, Jennifer Linn, Volker Puetz, Johannes C. Gerber

**Affiliations:** ^1^Institute and Policlinic of Neuroradiology, University Hospital Carl Gustav Carus, Dresden, Germany; ^2^Else Kröner-Fresenius Center for Digital Health, Dresden University of Technology, Dresden, Germany; ^3^Institute and Policlinic of Radiology, University Hospital Carl Gustav Carus, Dresden, Germany; ^4^Department of Neurology, University Hospital Carl Gustav Carus, Dresden, Germany

**Keywords:** basilar artery occlusion (BAO), endovascualar treatment, contact aspiration thrombectomy, complete recanalization, thrombus characteristics

## Abstract

**Objective:** To assess whether angiographic thrombus surface phenotype has an impact on efficacy of contact aspiration (CA) thrombectomy in patients with basilar artery occlusion (BAO).

**Methods:** From January 2016 to December 2019, consecutive stroke patients with a BAO and first-line CA were analyzed in this retrospective study. We assessed baseline and imaging characteristics and treatment and clinical outcomes. We rated thrombus surface phenotype on pre-treatment digital subtraction angiography in a three-reader-consensus setting. Primary outcome was complete recanalization (modified treatment in cerebral ischemia [mTICI] 3 and arterial occlusive lesion [AOL] 3) after first-line CA without additionally stent retriever passes. Data analysis was stratified according to thrombus surface phenotype and complete first-line recanalization.

**Results:** Seventy-eight patients met the inclusion criteria. Median age was 74 years (IQR 64–80), 64% were male, and median baseline NIHSS score was 24 (IQR 7–32). Thirty patients had a regular and 16 patients had an irregular thrombus phenotype. Thrombus surface was not assessable in 32 patients. In patients with a regular phenotype, complete recanalization was more often achieved compared to irregular and non-ratable phenotypes (50 vs. 18.8% and 21.9%; *p* = 0.027). Patients with a regular phenotype [odds ratio [OR] 8.3; 95% confidence interval [CI]: 1.9–35.8; *p* = 0.005], cardioembolic stroke (OR 12.1, 95% CI: 2.0–72.8; *p* = 0.007), and proximal end of the thrombus in the middle basilar artery segment (OR 5.2, 95% CI: 1.0–26.6; *p* = 0.046) were more likely to achieve complete recanalization after first-line CA without rescue therapy.

**Conclusion:** The efficacy of CA may differ according to the angiographic thrombus surface phenotype in patients with BAO. A regular phenotype is associated with higher rates of complete recanalization in first-line CA. However, assessment of thrombus phenotype is frequently not feasible in BAO.

## Introduction

Without successful recanalization, basilar artery occlusion (BAO) is associated with high morbidity and mortality (1). Endovascular thrombectomy (EVT) is the standard of care for anterior circulation large vessel occlusion (acLVO) demonstrating high rates of recanalization and favorable outcome (2). In contrast, randomized controlled trials have not confirmed a benefit of EVT and best medical management (BMM) compared with BMM alone for patients with BAO (3, 4).

Early and complete recanalization seems to be a major factor for a favorable clinical outcome in BAO patients (5–7). First-line contact aspiration (CA) thrombectomy may be associated with higher rates of complete recanalization and a shorter procedure time in BAO compared to stent retriever (SR) (7, 8). In patients with an acLVO, the presence of a regular thrombus surface on pre-treatment digital subtraction angiography (DSA) seems to be associated with higher recanalization rates if treated by first-line CA (9, 10). This association has not been shown for BAO. However, a recent study has demonstrated that meniscoid-like thrombus surface is associated with higher recanalization rates if treated with CA (11).

We aimed to evaluate factors associated with complete recanalization using CA for BAO. We hypothesized that a regular thrombus surface in pre-treatment DSA is positively associated with complete recanalization in first-line CA without additional SR passes.

## Materials and Methods

### Patients and Study Design

We included consecutive patients with acute BAO undergoing first-line CA thrombectomy within 24 h from symptom onset who presented to our center between January 2016 and December 2019.

We prospectively recorded patients' baseline and clinical characteristics (sex, age, comorbidities, vascular risk factors, onset, and severity of stroke: National Institutes of Health Stroke Scale [NIHSS] and intravenous thrombolysis [IVT]) in our institutional stroke register. Stroke etiology was assessed according to Trial of Org 10172 in Acute Stroke Treatment (TOAST) criteria (12): (1) cardioembolic, large vessel atherosclerosis of (2) vertebral artery (VA) with arterio-arterial embolism or (3) BA with local atherosclerotic occlusion and (4) other or undetermined etiology. We included patients with a pre-hospital mRS 0–2. We excluded patients with revascularization of BA in pre-treatment DSA and in whom no access to the target occlusion could be gained.

This study has institutional ethics committee approval (Ethikkommission TU Dresden; EK272072017) with waiver of informed consent and follows the Strengthening the Reporting of Observational Studies in Epidemiology (STROBE) reporting guideline (13).

### Procedural Data

We prospectively collected procedure times, endovascular devices used, number of device passes, and periprocedural complications. Primary CA was the preferred first-line treatment modality in our center for patients with BAO. We used different aspiration catheters (ACE 60, 64 or 68, Penumbra Inc., Alameda, CA, USA) that were combined with stent retrievers (SR) (EMBOTRAP II, Cerenovus, Johnson and Johnson, Raynham, MA, USA; pREset, phenox GmbH, Bochum, Germany; Separator 3D, Penumbra Inc., Alameda, CA, USA) if a rescue strategy was necessary. In case an underlying high-grade stenosis of the vertebral or basilar artery had to be treated, we used balloon-mounted or self-expanding stents (Coroflex Blue, B. Braun Melsungen AG, Berlin, Germany; PRO-Kinetic Energy Explorer, Biotronik AG, Bülach, Switzerland; Neuroform Atlas, Stryker Neurovascular, Fremont, CA, USA; Enterprise, Codman Neuro, Johnson and Johnson, Raynham, MA, USA) combined with balloon angioplasty, if needed. We selected the type of anesthesia (procedural sedation or general anesthesia) in cooperation with the anesthetist and stroke neurologist based on stroke severity, cardiopulmonary stability, and patient cooperation. The patients were treated on a biplane angiography system (AlluraXper FD20/15, Philips Medical Systems, Hamburg, Germany) and then transferred to the neurological intensive care unit or stroke unit.

### Occlusion Characteristics and Recanalization Status

**Figure 1:**
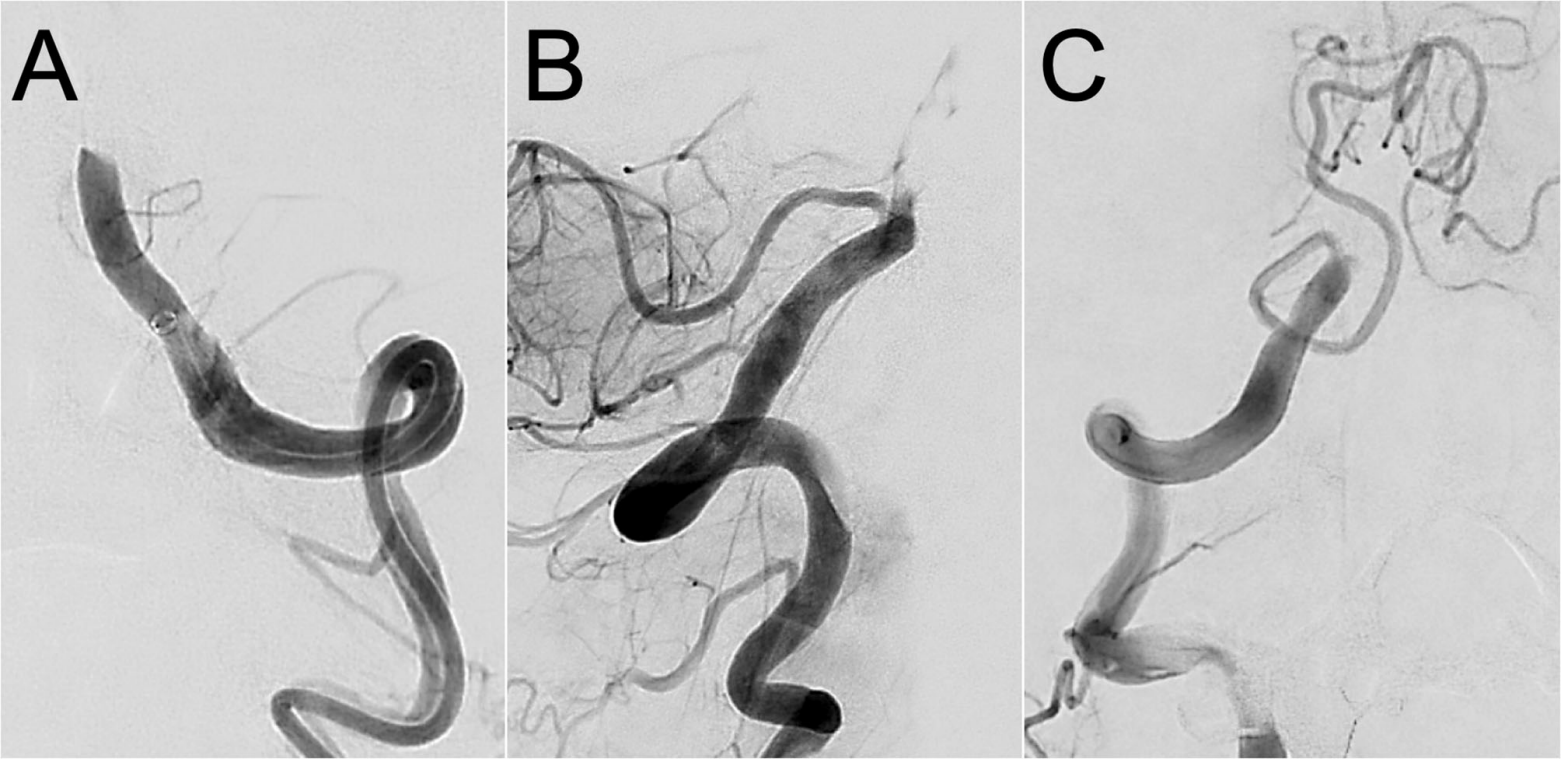
Different gradings of thrombus surface phenotype in basilar artery occlusion. Pre-treatment digital subtraction angiography images of a basilar artery occlusion in a patient with a regular **(A)** and irregular **(B)** thrombus surface phenotype. Thrombus surface phenotype was non-ratable in panel **(C)** due to outflow in the ipsilateral posterior inferior cerebellar artery (PICA).

Three readers retrospectively analyzed pre-treatment occlusion characteristics on computed tomographic or magnetic resonance angiography. For localization of the occlusion, we divided the VA, BA, and the posterior cerebral arteries (PCA) into segments, as widely accepted (14). We assessed the location of the proximal thrombus end and the number of involved vessel segments. Anatomical variants of the V4-segment of vertebral arteries (VA) were scored as described before (15). The readers independently categorized the proximal surface of the thrombus on the pre-treatment DSA as “regular” if the profile was smoothly straight, convex, or concave (i.e., meniscoid-like) in the full vessel diameter, and “irregular” if these criteria were not met (Figure 1) (9). If readers were unable to determine the phenotype, we categorized the thrombus surface as not assessable and stated a reason. Status of recanalization (modified Treatment In Cerebral Ischemia [mTICI]) and (Arterial Occlusive Lesion [AOL]) was determined on the final angiogram of the procedure (16). Discrepant cases were resolved by consensus of the three readers.

### Outcome Measures

The primary objective of our study was to evaluate the rate of complete recanalization (mTICI 3 and AOL 3) after first-line CA without additional SR passes depending on the phenotype of the thrombus surface. Apart from the occlusion phenotype, we analyzed the association of other variables (stroke etiology, thrombus location and extent, and anatomical variants of VA) with complete recanalization after first-line CA. Secondary outcome measures were favorable clinical outcome [modified Rankin Scale [mRS] 0–3] and death (mRS 6) after 90 days.

### Statistical Analysis

The study population was stratified according to the phenotype of the thrombus surface and complete first-line recanalization. Continuous variables are presented as median and interquartile range (IQR). Categorical variables are presented as absolute and relative frequencies. Distributions of continuous variables were tested for normality with the Kolmogorov–Smirnov test. We compared baseline characteristics, treatment and outcome variables between the groups using the chi-squared test and Fisher's exact test or an analysis of variance (ANOVA) and the Kruskal–Wallis test as appropriate. Logistic regression analyses were carried out on predictors identified by univariate analysis. The inter-rater reliability of grading the occlusion phenotype and recanalization status was assessed using Fleiss' Kappa (κ) with its 95% confidence interval (CI). Kappa values 0–0.2 indicate slight, 0.21–0.4 fair, 0.41–0.6 moderate, 0.61–0.8 substantial, and 0.81–1.00 almost perfect agreement (17). For all statistical analyses, a *p* < 0.05 or its Bonferroni correction was considered significant. We analyzed the data with SPSS Statistics (Version 25, IBM, Armonk, USA).

## Results

### Patients

**Figure 2:**
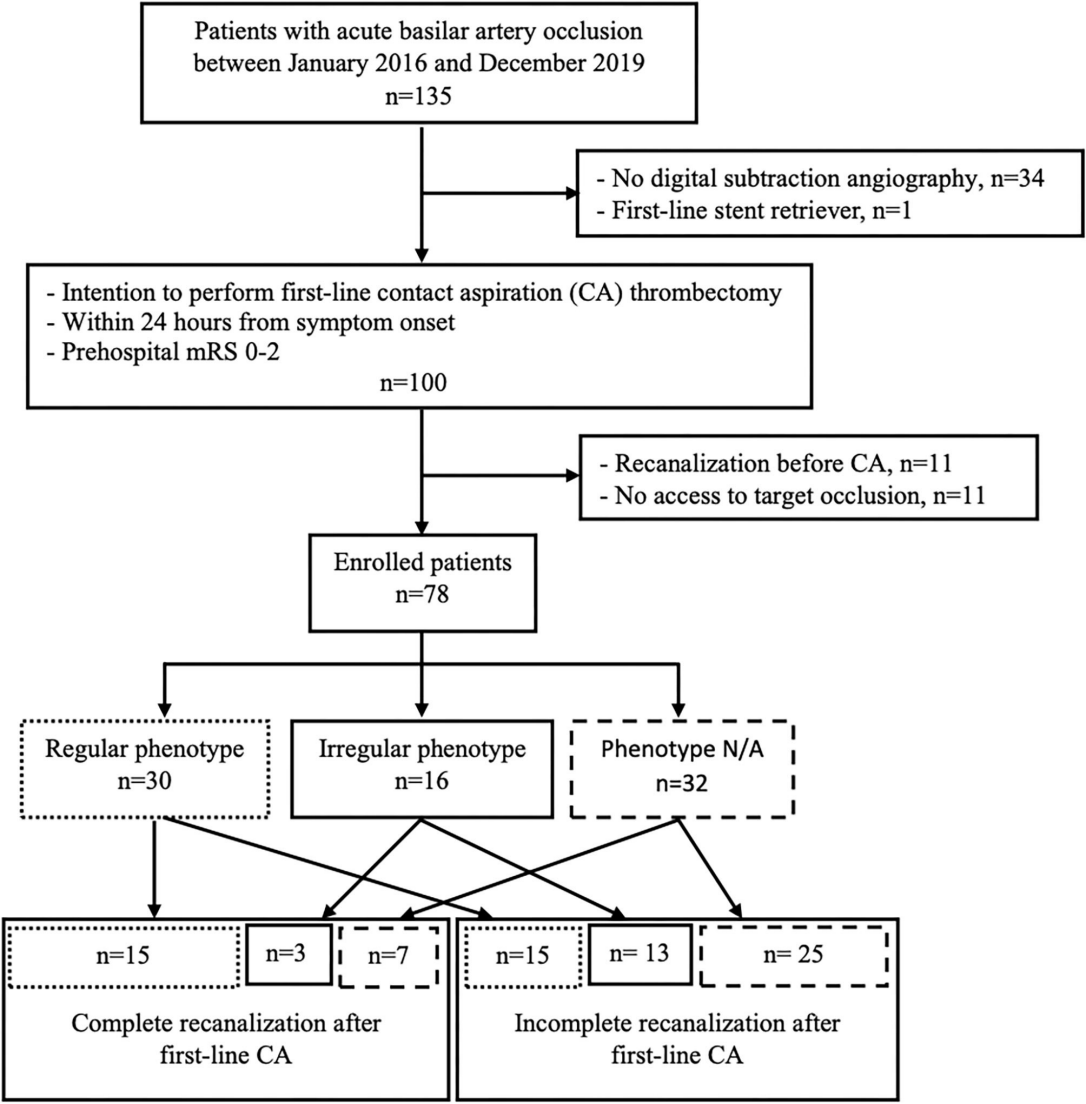
Flow chart of patient selection. CA, contact aspiration; N/A, not applicable; mRS, modified Ranking Scale.

Of 135 patients with BAO, we excluded 34 patients who did not qualify for DSA due to large infarct size (*n* = 2), recanalization of BAO on CT-angiography in our hospital (*n* = 28), study randomization (*n* = 1), and neurological improvement to NIHSS 0 (*n* = 3). One patient was treated with a stent retriever first-line. Of 100 patients that received a digital subtraction angiography (DSA) with the intention to perform first-line CA, we subsequently excluded 11 patients with recanalized BA on pre-treatment DSA and 11 patients in whom we could not access the target arterial occlusion. Finally, we included 78 patients in the study (Figure 2). Median age was 74 years (IQR 64–80) and 64% were of male sex. The median NIHSS on admission was 24 (IQR 7–32). Overall, 40 (51.3%) patients received IVT before proceeding to endovascular treatment. The median time from symptom onset to groin puncture was 295 min (IQR 185–365 min).

### Thrombus Surface Phenotype

We rated 30 patients as having a regular and 16 patients as having an irregular phenotype. Phenotype grading was not possible in 32 patients due to poor delineation of the thrombus surface because of the inflow from the contralateral vertebral artery (*n* = 19), early outflow *via* cerebellar arteries (*n* = 2), or a stenosis of the intracranial VA or BA (*n* = 11). The inter-rater reliability of rating the phenotype of the occlusion was moderate with κ = 0.46 (95% CI: 0.36–0.55).

If the proximal end of the thrombus was located in the distal segment of the BA, we were able to rate its surface significantly more often than in other occlusion locations (3.7% N/A ratings in distal vs. 40.7% in middle, 22.9% in proximal segment of BA, and 33.3% in V4; *p* < 0.05). We found no association of stroke etiology with thrombus surface phenotype (*p* = 0.589) but a non-significant trend to occlusion location (*p* = 0.055).

### Procedural Data and Recanalization Results According to Thrombus Phenotype

Stratified according to the phenotype of the thrombus surface, patients were comparable regarding baseline characteristics, procedural data, and clinical outcome (Table 1). We used SR as a rescue strategy in one patient with regular, in five with irregular, and in four patients with undetermined phenotype. Stroke etiology, thrombus length, and rates of hypo-/aplastic V4-segments did not differ between the phenotypical groups.

**Table 1:** Baseline and procedural characteristics, treatment results, and outcomes.

	**Overall, *n* = 78**	**Phenotype**			
		**Regular, *n* = 30**	**Irregular,** ***n*** **= 16**	**N/A,** ***n*** **= 32**	***p*** **-value**
**Baseline and clinical data**					
Age, years, median (IQR)	74 (64–80)	73 (63–80)	76 (71–81)	74 (65–80)	0.483
Men, *n* (%)	50 (64)	22 (73)	9 (56)	19 (59)	0.570
Hypertension, *n* = 72 (%)	52 (72.2)	19 (65.5)	13 (81.3)	20 (74.1)	0.561
Diabetes mellitus, *n* = 72 (%)	33 (45.8)	11 (37.9)	7 (43.8)	15 (53.6)	0.313
Dyslipidemia, *n* = 33 (%)	6 (18.2)	1 (5.9)	3 (50)	2 (20)	0.108
Atrial fibrillation, *n* = 73	32 (43.8)	12 (41.4)	7 (43.8)	13 (46.2)	0.797
Previous stroke, *n* = 73 (%)	14 (19.2)	4 (13.8)	4 (25)	6 (21.4)	0.597
Coronary artery disease, *n* = 33 (%)	1 (3)	1 (5.9)	0	0	0.708
Antithrombotic treatment, *n* (%)	58 (74.4)	22 (73.3)	13 (81.3)	23 (71.9)	0.489
Drip and ship, *n* (%)	48 (61.5)	17 (55.7)	10 (62.5)	21 (65.6)	0.505
Baseline NIHSS, median (IQR)	24 (7–32)	26 (7–32)	19 (11–28)	26 (7–32)	0.815
Prehospital mRS 0–2, *n* (%)	78 (100)	30 (100)	16 (100)	32 (100)	–
IVT, *n* (%)	40 (51.3)	15 (50)	6 (37.5)	17 (53.1)	0.612
Onset to groin puncture, min, *n* = 52	295 (185–365)	251 (201–328)	280 (183–410)	287 (216–326.5)	0.248
**Procedural data**					
General anesthesia, *n* (%)	71 (91)	27 (90)	16 (100)	28 (87.5)	0.286
Duration of procedure, min, mean (IQR)	55 (37–93)	34 (52–76)	56 (36–94)	63 (35–103)	0.897
CA passes, median (IQR)	1 (1–2)	1 (1–2)	1 (1–2)	1 (1–1)	0.586
SR rescue, *n* (%)	10 (12.8)	1 (3.3)	5 (31.3)	4 (12.5)	0.323
Angioplasty extracranial, *n* (%)	10 (12.8)	3 (10)	2 (12.5)	5 (15.6)	0.363
Angioplasty intracranial, *n* (%)	7 (9)	4 (13.3)	0	3 (9.4)	0.430
Procedural complication, *n* (%)	3 (3.8)	0	2 (12.5)	1 (3.1)	0.092
**Recanalization and outcome**					
Complete recanalization, *n* (%)	25 (32.1)	15 (50)	3 (18.8)	7 (21.9)	**0.027**
Final substantial recanalization, *n* (%)	67 (85.9)	27 (90)	12 (75)	28 (87.5)	0.358
90-day mRS 0–3, *n* = 72 (%)	26 (36.1)	9 (32.1)	5 (33.3)	12 (41.4)	0.744
90-day mRS 6, *n* = 72 (%)	31 (43.1)	10 (35.7)	7 (46.7)	14 (48.3)	0.567

*CA, contact aspiration; IVT, intravenous thrombolysis; IQR, interquartile range; min, minute; mRS, modified Ranking Scale; N/A, not applicable; NIHSS, National Institutes of Health Stroke Scale; SR, stent retriever. Bold values are statistical significance*.

A complete recanalization after first-line CA without additional SR passes was achieved in *n* = 15/30 (50%) of patients with a regular surface compared to *n* = 3/16 (18.8%) of patients with an irregular phenotype and *n* = 7/32 (21.9%) of patients where thrombus surface characteristics could not be determined (*p* < 0.05). In total, *n* = 67 (85.9%) of the patients had a substantial reperfusion (mTICI 2b-3) after all devices with no group differences according to the phenotype of the occlusion site. The inter-rater reliability for mTICI and AOL ratings were substantial with κ = 0.67 (95% CI: 0.57–0.77) and κ = 0.77 (95% CI: 0.69–0.85), respectively. Three patients experienced complications: two hemorrhages (one subarachnoid and one intracerebral hemorrhage) and one embolization to a new territory (PCA territory).

### Predictors of Complete First-Line Recanalization

Patients with a complete recanalization (mTICI 3 and AOL 3) after first-line CA without additional SR passes more often had a regular thrombus phenotype and cardioembolic stroke and less frequently arterio-arterial embolism as stroke etiology. The location of the proximal thrombus end was less frequently in the V4-segment of the VA and more frequently in the middle segment of the BA compared to patients without complete recanalization (Table 2). The number of involved vessel segments was lower in successful recanalized patients.

**Table 2:** Predictors of complete first-line recanalization.

	**Complete first-line recanalization**		
	**Yes,** ***n*** **= 25**	**No,** ***n*** **= 53**	***p*** **-value**
**Phenotype, n (%)**			
Regular	16 (60)	15 (28.3)	**0.012**
Irregular	3 (12)	13 (24.5)	0.164
N/A	7 (28)	25 (47.2)	0.108
**Stroke etiology, n (%)**			
VA arterio-arterial embolism	3 (12)	19 (35.8)	**0.024**
BA atherosclerosis	1 (4)	10 (18.9)	0.072
Cardioembolism	19 (76)	18 (34)	**0.001**
Undetermined or other	2 (8)	6 (11.3)	0.496
**Clot location, n = 69 (%)**			
V4	2 (8.3)	15 (33.3)	**0.021**
BA proximal	4 (16.7)	12 (26.7)	0.282
BA middle	13 (54.2)	11 (24.4)	**0.012**
BA distal	5 (20.8)	7 (15.6)	0.390
Involved segments, median (IQR)	2 (2–2)	2 (1–3)	**0.002**
VA contralateral hypo-/aplastic, *n* (%)	17 (68)	29 (60)	0.614
**Outcome 90 days, n = 72 (%)**			
mRS 0–3	47.8 (11)	30.6 (15)	0.192
mRS 6	5 (21.7)	26 (53.1)	**0.011**

*BA, basilar artery; mRS, modified Ranking Scale; N/A, not applicable; VA, vertebral artery; V4, distal segment of vertebral artery. Bold values are statistical significance*.

In logistic regression analysis, patients with a regular phenotype [odds ratio [OR] 8.3, 95% CI: 1.9–35.8; *p* = 0.005], cardioembolic stroke (OR 12.1, 95% CI: 2.0–72.8; *p* = 0.007), and proximal end of the thrombus in the middle BA segment (OR 5.2, 95% CI: 1.0–26.6; *p* = 0.046) were more likely to reach complete first-line CA recanalization. The logistic regression model was significant [chi-squared test (6) = 29.615; *p* < 0.001] and showed a large effect size (*f*^2^ = 0.93).

### Clinical Outcome

Clinical outcome at 90 days was available for 72 patients. In total, 26 (36.1%) patients had a favorable outcome and 31 (43.1%) died. Patients with complete recanalization after first-line CA had a non-significant trend to better outcomes (*n* = 11/23 vs. *n* = 15/49; n.s.) and a significantly lower mortality rate (*n* = 5/23 vs. *n* = 26/49; *p* < 0.05).

## Discussion

We found that a regular thrombus phenotype, cardioembolic stroke etiology, and occlusion in the middle segment of the BA might be predictors of complete recanalization if CA is chosen as the first-line thrombectomy method in BAO patients. However, phenotyping of the thrombus surface in BAO was technically less feasible compared to anterior circulation large vessel occlusions (acLVO) (9, 18).

Early complete recanalization seems to be a strong predictor for better clinical outcomes in BAO and is the primary goal of EVT regardless of the technique used (5–7). In BAO, first-line CA thrombectomy may be associated with higher rates of complete recanalization compared to SR (7, 8). The finding that a regular thrombus surface impacts on first-line CA recanalization success confirms our hypothesis and is in line with previous findings in acLVO (9, 10). However, we have no data on the impact of the thrombus surface phenotype on first-line SR effect as we used first-line SR only in one patient with BAO during the study period. Additionally, the rates of SR rescue strategy were too small for further subgroup analyses.

Recently, another study suggested the “meniscus sign” for the angiographic phenotype of the BAO site. In this study, patients were treated with both techniques, CA and SR (11). The meniscus sign, partly resembling our definition of a regular thrombus phenotype, was associated with higher complete recanalization rates in CA compared with SR after propensity score matching (11). These results underline our hypothesis that the efficacy of EVT technique may differ according to the angiographic phenotype in BAO. There are different definitions of the type of thrombus surface phenotype that best responds to CA (9–11). We would recommend our definition of a “regular” phenotype (i.e., smoothly straight, convex, or concave profile in the full vessel diameter) for clinical practice as it unifies the different classifications and a stricter definition could exclude patients who might benefit from a phenotype-based device selection.

Cardioembolic stroke and more distal thrombus location were associated with higher rates of complete revascularization. This is in line with previous studies but does not apply to CA only as it was described in patients treated by SR or IVT as well (8, 19, 20).

We found no association of the thrombus phenotype with stroke etiology. This is in contrast to a previous study showing an association of the positive clot meniscus sign with an embolic etiology (21). Hypothetically, the effectiveness of CA in recanalizing occlusions with a regular thrombus surface might depend on the consistency of the thrombus rather than the underlying stroke etiology. Histological thrombus composition seems to be a key factor in determining susceptibility to mechanical manipulation and the degree of successful reperfusion (22). Future studies are needed to assess the association of thrombus phenotype, composition, and stroke etiology.

The inter-rater reliability for mTICI and AOL scales in BAO were substantial. We used a combination of mTICI and AOL to assess complete revascularization as there is controversy as to which revascularization scale is the most suitable for posterior circulation stroke (23).

In line with previous studies (5–7), complete first-line revascularization was associated with better clinical outcomes; however, our study was not powered to draw any firm conclusion. Prospective studies are required to assess whether thrombus surface characteristics should influence the choice of the revascularization technique used in BAO to achieve best procedural and clinical outcomes. Choosing the most effective first-line thrombectomy device can also have economic benefits as it avoids the need for additional devices.

A limitation of thrombus surface phenotyping in BAO is the moderate inter-rater agreement compared to substantial agreement in acLVO (9, 10, 18). We introduced a third category “not applicable” if the thrombus surface phenotype could not be determined. We outlined two main reasons for this difference between acLVO and BAO: First, the indirect delineation of the thrombus surface against the contrast medium was interfered by inflow from the contralateral vertebral artery or early outflow *via* cerebellar arteries. Second, intracranial vertebral or basilar artery stenosis disguised the thrombus surface. Interestingly, low flow conditions due to hypoplastic or aplastic contralateral VA or the absence of a local stenosis in BA were not associated with higher rates of successful phenotyping. Instead, phenotyping was most successful, if the occlusion occurred in the distal segment of the BA. Based on the study focus, we excluded BAO patients with no DSA or no access to target arterial lesion and patients with recanalized BA before EVT. This study design could affect the results of our analysis. However, thrombus surface analysis proved very difficult in CTA and MRA in preparatory studies.

This single-center study has all the limitations of a retrospective observational design. However, we prospectively recorded the data in our institutional stroke register and a single-center study offers homogeneity in (peri-) procedural patient care. Another limitation is the small number of patients and differing experience of the interventionalists that both might be unaccounted confounding variables.

## Conclusion

The efficacy of CA thrombectomy may differ according to the angiographic phenotype of the thrombus surface at the target occlusion in patients with BAO. A regular phenotype may be an independent predictor for complete recanalization with first-line CA. Thrombus phenotyping in pretreatment DSA may be used to assist in choosing the first-line thrombectomy strategy. However, thrombus phenotyping was often not feasible in our cohort of BAO patients.

## Data Availability Statement

The raw data supporting the conclusions of this article will be made available by the authors, without undue reservation.

## Ethics Statement

The studies involving human participants were reviewed and approved by Ethikkommission TU Dresden. The ethics committee waived the requirement of written informed consent for participation.

## Author Contributions

DK and JG contributed to conception and design of the study. MG, PK, JL, DK, JG, L-PP, and VP had a major role in data acquisition. KH and DK organized the database. KH, RW, PK, JG, VP, and DK contributed to the analysis of the data. DK performed the statistical analysis and wrote the first draft of the manuscript. All authors contributed to manuscript revision, read, and approved the submitted version.

## Conflict of Interest

JG has received speaking fees from Penumbra Inc. The remaining authors declare that the research was conducted in the absence of any commercial or financial relationships that could be construed as a potential conflict of interest.

## Publisher's Note

All claims expressed in this article are solely those of the authors and do not necessarily represent those of their affiliated organizations, or those of the publisher, the editors and the reviewers. Any product that may be evaluated in this article, or claim that may be made by its manufacturer, is not guaranteed or endorsed by the publisher.
